# The Tousled-Like Kinases as Guardians of Genome Integrity

**DOI:** 10.5402/2012/627596

**Published:** 2012-05-20

**Authors:** Arrigo De Benedetti

**Affiliations:** Department of Biochemistry and Molecular Biology and Feist-Weiller Cancer Center, Louisiana State University Health Sciences Center, 1501 Kings Highway, Shreveport, LA 71130, USA

## Abstract

The Tousled-like kinases (TLKs) function in processes of chromatin assembly, including replication, transcription, repair, and chromosome segregation. TLKs interact specifically (and phosphorylate) with the chromatin assembly factor Asf1, a histone H3-H4 chaperone, histone H3 itself at Ser10, and also Rad9, a key protein involved in DNA repair and cell cycle signaling following DNA damage. These interactions are believed to be responsible for the action of TLKs in double-stranded break repair and radioprotection and also in the propagation of the DNA damage response. Hence, I propose that TLKs play key roles in maintenance of genome integrity in many organisms of both kingdoms. In this paper, I highlight key issues of the known roles of these proteins, particularly in the context of DNA repair (IR and UV), their possible relevance to genome integrity and cancer development, and as possible targets for intervention in cancer management.

## 1. General Information on Tousled-Like Kinases

The *Tousled* locus was originally identified in *A. thaliana* and *Antirrhinum majus *during a study of mutations leading to defects in meristem expansion. Mutations of *Tousled* produce a complex phenotype characterized by specific defects in development of leaf and floral organs [1]. This was proposed to be linked to a replicative defect during organogenesis, but it may also result from failure to protect the genomefrom DNA damage [2, 3], resulting in developmental aberrations [4, 5]. Highly related* Tousled*-like genes can be found in many organisms in both kingdoms, several of which encode multiple transcripts resulting in different protein isoforms [6]. It was originally proposed that *Tousled* (TSL) may be a component in a signal transduction pathway controlling cell proliferation and DNA synthesis during organogenesis, and this immediately prompted a search for its substrates. However, unlike most kinases that usually display a propensity to phosphorylate numerous substrates, after many years of study, only a few direct “interacting” substrates of TLKs have been identified, namely, the histone chaperone Asf1 [7], histone H3-S10 [8], Aurora B [5], and more recently Rad9 [9]. This suggested a function for TLKs in chromatin assembly [9, 10], during transcription [2, 11], DNA repair [3, 9, 12], and condensation of chromosomes at mitosis [4, 5]. The latter function, which was found critical for proper chromosome segregation, prompted a search for additional “indirect” substrates and functions and resulted in the identification of an activity on myosin II in mammalian cells [13] and on the chromosome passenger complex in trypanosomes [14]. The search for TLKs functions at mitosis and meiosis is currently a very active pursuit in several labs in more genetically tractable organisms like *Drosophila* [15] and *C. elegans* (Jill Schumacher, personal communication). In addition, whereas only nuclear functions were initially proposed for these proteins, some splice variants localize also to the cytoplasm [8], perhaps due to their reported interaction with 14-3-3 proteins [16] with their shuttling function and hence could play additional roles in potential cytoplasmic substrates, one of which was identified as the DEAD-box p68 RNA helicase [17]. More emphasis is presented next for three of the most important substrates of TLKs: Asf1, Rad9, and histone H3.

### 1.1. The Chromatin Assembly Factor Asf1

 Asf1 is a histone H3-H4 chaperone [18] that is essential in mammals [19] and other organisms [20, 21] but not in *S. cerevisiae*, although such cells deleted for Asf1 are sensitive to genotoxins [22]. A recent review on Asf1 and other histone chaperones can be found in [23] and its critical importance for epigenome maintenance in [24]. Asf1, in conjunction with another chaperone called CAF1, promotes the assembly of nucleosomes onto newly replicated DNA, but it can also promote nucleosome eviction at activated promoters [25–27]. Thus, Asf1 is generally involved in chromatin remodeling, which also entails DNA repair [22, 28]. The crystal structure of Asf1 in complex with H3-H4 was solved at high resolution [29], and Asf1 was found to cover the dorsal side of the H3-H4 dimer, thereby sterically preventing formation of the core tetramer. This is thought to be important for disrupting nucleosomes during transcription [30] or remodeling of chromatin in damaged DNA [3, 9], and the role of TLK1B in radioprotection was initially attributed to its effect on Asf1, presumed to be via its phosphorylation [12]. More recently, however, I showed that TLK1B can stimulate chromatin assembly in vitro in conjunction with Asf1 regardless of its phosphorylation [31]. This suggested that TLK1/1B act as chaperones in chromatin assembly, in addition to their kinase function. Hence, an important role of TLKs via Asf1 is to promote nucleosomes eviction at DSBs and access of the repair machinery to unencumbered DNA.

### 1.2. Rad9

 Rad9, Rad1, and Hus1 form a trimeric complex (termed 9-1-1) that is structurally similar [32, 33] to the PCNA “sliding-clamp,” which encircles the DNA conferring processivity to polymerases [34–36]. 9-1-1 assembles in a complex at sites of damage [37], and it is the genotoxin-activated RFC-Rad17 “clamp loader” that locks 9-1-1 onto DNA [37]. The 9-1-1 may then serve as a scaffold for assembly of DNA repair proteins, Flap endonuclease [38, 39], DNA polymerase *β* [40], DNA ligase 1 [41], and DNA glycosylase MutY [42], in addition to aiding processing of the DNA ends by its own exonucleolytic activity [43–45]. We showed that TLK1B phosphorylates Rad9 at S328 and that this appears to play a key role in resumption of the cell cycle arrested after IR. However, TLK1B also had a function as a chaperone for Rad9 assembly at DSBs that was independent of its kinase function [9]. A possibility is that the regulated binding of 9-1-1 and TLK1B to DSBs recruits repair enzymes and a chromatin disassembly apparatus to facilitate access to unencumbered DNA and promote efficient DSB repair [9], and only subsequently in the DNA damage response (DDR) disengagement and deactivation of the checkpoint [46]. Rad9 participates in additional functions of the DDR and in repair and also in restart of stalled replication forks, along with numerous other proteins, like RHINO and TopBP1 [47] or WRN [48]. Although the Rad9 C-terminal tail (119 aa) shares no homology with PCNA and is thought to be nonessential for the formation of the 9-1-1 complex [35], this region is multiply phosphorylated, constitutively and inducibly in response to genotoxic stress [49, 50]. Rad9 is normally phosphorylated independently of the cell cycle at S277, S328, S336, T355, and S387 [49]. Cell-cycle-dependent phosphorylation of Rad9 at Thr 292 occurs during mitosis in a Cdc2-dependent manner [49]. Moreover, Rad9 is intensely phosphorylated in response to DNA damage. Although damage-dependent phosphorylation of Rad9 was initially believed to modulate the stability of the 9-1-1 complex [51], it is now believed that neither constitutive nor damage-induced phosphorylation influences the interaction of Rad9 with its partners Rad1 and Hus1 [37, 49]. Most studies point to the role of damage-activated Rad9 phosphorylation in downstream cell signaling via activation of Chk1, and it was reported that phosphorylation of Rad9 influences cell viability after UV and hydroxyurea cell-cycle stalling [50]. Substitutions at all carboxy-terminal 8 phosphorylation sites compromised Rad9 interaction with TopBP1 and impaired the cellular response to DNA damage [49]. Collectively, these results suggest that Rad9 phosphorylation regulates protein interactions and downstream cell signaling from DNA damage. TLKs are the only known kinases that specifically phosphorylate Rad9-S328. Phosphorylation at S328 was determined as a prerequisite for additional phosphorylation of Rad9 [52]. Our studies with reconstituted Rad9−/− cells indicated that S328 phosphorylation is not essential for Rad9 interaction with Hus1 and Rad1 [9], consistent with previous studies [37]. However, phosphorylation at S328 appeared to be important for exiting cell-cycle arrest after production of DSBs and resulting in a smaller fraction of apoptotic cells and better clonogenic survival [9]. Some studies have implicated stress-activated Rad9 phosphorylation to activation of Chk1 [50, 53], which mediates the cell cycle checkpoint. Indeed, current research in our lab has shown that inhibiting TLKs with specific chemical inhibitors results in incapacity to exit the cell cycle checkpoint and high rates of apoptosis when combined with DSB-inducing agents (to be published elsewhere). We observed similar results by overexpression of a TLK kinase-dead (KD) and observed a delay in the release of the Rad17-clamp-loader and Rad9 from a single genomic DSB introduced with the HO nuclease transiently expressed from Adenovirus [46]. The most logical conclusion arising from these results, and from the specific pattern of activity of TLKs (see below), is that the S328 phosphorylation of Rad9 by TLKs is critical for deactivation of the DDR checkpoint following DNA repair.

### 1.3. Histone H3

 Phosphorylation of histone H3 at Ser10 was recognized as the first substrate of TLK1B [8], as demonstrated both biochemically and by direct mass-spec measurements; this was further confirmed by genetic complementation of a yeast strain defective in Ipl1 (Aurora kinase of *S. cerevisiae*) that is the main H3-S10 kinase in this organism [8]. The significance of this phosphorylation remains unclear. *Tousled* (TSL) could phosphorylate histone H3 in vitro, just like mammalian TLK1B, but the recombinant *C. elegans* TLK was not effective at phosphorylating directly H3 but was highly stimulatory in conjunction with Air-1 (Aurora Kinase) [5], which was found to be an interacting target of the single TLK protein in *C. elegans* [5]. Phosphorylation of H3 by Aurora kinase is a hallmark of mitosis, and that specific phosphorylation is proposed to be critical for chromosome condensation at mitosis [54]. In that context, it is not clear if the role of TLK-mediated phosphorylation of H3 is central to mitosis, although the phosphorylation of H3-S10 is reduced in nonsynchronized cells expressing the KD, and the condensation of chromosomes and phosphorylation of H3 is reduced at mitosis [4]. But this of course could be also an indirect effect on Aurora kinase activity [5, 55]. More importantly, however, inhibition of TLK activity by genotoxic stress (see below) by either IR or UV results in reduced levels of H3-S10 in unsynchronized cells [3, 8]. Since mitotic cells represent only a small minority of cycling cells, even the large ~5-fold increase in H3P(S10) seen during mitosis would account for only a very small amount of the H3P from the total population. Hence, it would seem logical to assume that TLK-mediated H3P phosphorylation probably accounts for some other function in chromatin maintenance. If for example TLK1/1B is the main H3 kinase involved in a “chromosomal response” to DNA damage, then ATM-mediated inhibition of TLK1 (see below) is expected to result in a loss of H3 phosphorylation by endogenous phosphatases and in altered kinetics of chromatin assembly during replication and/or repair. It is possible that physiologically the increased TLK1B synthesis following IR [56] can help offset the loss of TLK activity resulting from IR and restore appropriate levels of H3P later on during the recovery. The reduction of H3P following genotoxic stress (IR) was previously reported also by another group [57]. In any case, there are other situations where H3-S10 phosphorylation is induced beside mitosis, and a clear case is that of the “nucleosomal response” at the early-response genes following mitogenic stimulation. Gene disruption in murine embryonic stem cells, and genetic evidence from Coffin-Lowry syndrome, has implicated Rsk-2 as the kinase directly responsible for phosphorylating H3 following mitogenic stimulation [58]. On the other hand, another kinase (MSK1) was reported to phosphorylate H3 more efficiently and be sensitive to the kinase inhibitor H89, which impairs the nucleosomal response, whereas Rsk-2 was insensitive to this inhibitor [59]. However, it now seems clear that several families of H3-S10 kinases exist (e.g., Ipl1/Aurora and NIMA) and may be involved in different or partially overlapping functions [60, 61]. We have clearly shown that recombinant TLK1B phosphorylates H3 at S10 and could complement a temperature-sensitive mutant of Ipl1 in yeast and restore H3P in those cells at the nonpermissive temperature. Furthermore, it could do so with high specificity in a mix of core histones, and in cells overexpressing TLK1B we found increased levels of H3 phosphorylation [8]. These findings, as well as the genetic complementation data, strongly suggest the inclusion of the *Tousled* family of kinases to the list of H3-S10 kinases, even though their precise role during the cell cycle or perturbations of it (inhibitory or stimulatory) has not been fully elucidated. The use of newly identified specific chemical inhibitors of TLKs could perhaps shed light on the role of TLK-mediated phosphorylation of H3-S10 and its significance in chromatin assembly during normal division or after DNA damage.

## 2. TLKs in Man, as Guardians of Genome Stability, and Their Possible Involvement in Cancer

The first human TLK cDNA to be cloned, what we later referred to as the TLK1B splice variant (KIAA0137), was first identified during the random cloning of novel cDNAs from the human myeloid cell line KG-1 [62]. The cDNAs for TLK1 (Chr 2) and TLK2 (Chr 17) were later cloned during a PCR-based search for human kinases [63] and independently from an expression library screened on the basis of autophosphorylation activity ([64]; named PKU*β* and PKU*α* by these authors). Instead, we have independently cloned the TLK1B splice variant with a completely different screen, based upon polysomal redistribution of weakly translated transcripts that become preferentially recruited upon overexpression of eIF4E [8]. We subsequently found that TLK1B is synthesized efficiently in several cell lines overexpressing the translation factor/oncogene eIF4E, and we then presented several lines of evidence to confirm its translational regulation, particularly after genotoxic stress [56]. The significance of this translational regulation is discussed later when I emphasize the role of TLKs in DNA repair and protection from genotoxic agents, including IR and UV. Below, we propose that an important role for TLKs is as guardian of the genome, and we implicate a function in cancer development and progression. This derivation seems obvious given their role both in basic aspects of chromatin assembly, transcription, replication, and repair and also for their distinct role in chromosome segregation into daughter cells.

A high percentage of human tumors, including cancer of the prostate (CaP) and breast (BCA), show mutations in DNA repair genes and checkpoint functions that make them overly dependent on alternative pathways for survival. Unfortunately, this can result in carcinomas that are highly resistant to radiation therapy (XRT) or radiomimetic therapy (RMT) from failsafe repair mechanisms also designed to contain excessive genomic instability. Targeting those mechanisms can result in highly specific and effective therapies. We propose that the addition of inhibitors of TLKs to enhance response to radiochemotherapy will greatly benefit CaP and BCA patients' therapy management. In fact, ameliorating the effects of standard therapy, and possibly reducing its doses while maintaining specific killing, still seems to be the one of most promising course of action for the near future. Certainly, the success of PARP inhibitors for triple negative BCA seem to point in that direction [65].

To recapitulate some basic information before addressing TLKs in humans, the TLKs are involved in chromatin assembly, DNA repair, transcription, and chromosome segregation ([9] and references therein). Two TLK genes (TLK1 and TLK2) with several splice variants have been identified in humans [63]. TLK1/1B interacts specifically with the chromatin assembly factor Asf1 and Rad9 [9, 46], and we have presented evidence that TLK1B promotes repair by processing of the double-strand break (DSB) ends and disassembly of chromatin near the DSB to facilitate recruitment of repair proteins [9]. Since Rad9 is a critical mediator of the response to DNA damage, DDR checkpoint, and in repair (specifically of DSBs), it seemed that the TLK1-Rad9 interaction would be very important in implementing the mechanism of TLK1B-mediated radioprotection. The past few years have witnessed significant advances in understanding the roles of TLKs in the DDR [66] and in direct repair of DSBs [9], as well as their clinical relevance. In BCA, elevated expression of the TLK1B splice form is found in ~30% of the patients [67] and often corresponds to poor response to XRT [68] and doxorubicin [67], presumably due to efficient repair of DSBs in the tumor cells. We postulated that its expression could serve as a marker for prognosis as well as a target for therapeutic intervention. In addition, there are BCA cases where TLK1/1B is not elevated, but it is TLK2, which lies in a region of Chr 17q23 nearby the BRCA1 locus, that is amplified and/or overexpressed in a significant number of BCA specimens [69, 70]. Thus, for a large proportion of sporadic BCA, specific TLK inhibitors should be extremely beneficial as radio-chemosensitizers. The fact that TLKs are overexpressed likely renders tumor cells more dependent on these kinases than normal tissues and, hence, their preferential TLK-targeted killing. In contrast to BCA, in the most common human CaP cell lines, only one or the other TLK gene is expressed [71], although typically at high level—we do not have the story yet for the analysis of patient samples. The significance of the TLK/Rad9 axis is perhaps even greater for prostate cancer for which several studies have implicated the critical role of Rad9 in disease progression and prognosis. In one study, Rad9 was associated with tumor stage and was reported to regulate tumor growth in mice [72]. In another, the investigators found that Rad9 contains androgen-responsive elements and that its expression is also androgen regulated [73]. In a third study, Rad9 acted as a corepressor of AR transactivation [74]—all of which suggest that Rad9 expression may be a significant part of the “androgen switch” that leads to cancer cell survival and that Rad9 has functions beyond DNA repair that make it clinically relevant as a biomarker or in tumor growth control [72]. Additional implications for the role of Rad9 in CaP and other common cancers are reviewed in [75, 76]. Correspondingly, elevated expression of TLKs (along with Rad9) may be a significant marker of radioresistance in CaP cell lines and likely in cancerous samples and hence represents a hallmark of poor prognosis.

In contrast to Rad9, there is no report for the direct involvement of either of the two human Asf1 genes in cancer development, perhaps due to the critical importance of these histone H3/H4 chaperones for all mammalian cells (normal and cancer). Nonetheless, a recent report correlated the expression of Asf1b in prediction of BCA relapse, perhaps due to its higher importance for cell proliferation and chromosomes duplication [77].

It is noteworthy that translocations/amplifications involving 17q23 that include the TLK2 gene are not unique to BCA but are also found neuroblastomas [78] and glioblastomas multiforme [79].

## 3. TLKs in DNA Repair and as Possible Targets for Gene/Molecular Therapy

The fact that overexpression of TLKs (or more specifically the TLK1B splice variant) conferred a high degree of protection against IR in mouse cells was one of the first effects reported for these proteins [8]. It was soon found that the protection involved increased and more efficient repair of DSBs in living cells [9, 12], and then more precisely with in vitro plasmid repair assays with defined components and recombinant proteins [46]. In such reactions, the assembly of nucleosomes on the plasmid was simultaneously monitored as a decrease in the linking number via formation of high-mobility topoisomers in conjunction with repair of a DSB [46] or of excision of UV-induced pyrimidine dimers [3]. Hence, the specific contribution of Asf1 to repair of DSBs or UV damage could be studied in those conditions. For repair of the DSB, depletion of Asf1 had some effect on supercoiling, but it had only modest effect on religation of the ends. Quantitative analysis showed that conversion of the linear form to circular/relaxed and then supercoiled was complete after 20 min in control extract, but not until 40 min in Asf1-depleted. Hence, Asf1 albeit likely involved, was not essential for these repair reactions nor for supercoiling [46], at least for the case of cohesive-ends repair. Similarly, repair of UV-damaged plasmids did not absolutely depend on Asf1, although the kinetics of repair were strongly delayed [3], consistent with a previous report that looked at the contribution of Asf1/CAF1, and even TLKs, in repair of UV-damaged plasmids [80]. The identification of Asf1 [7] and later Rad9 [9] as two main targets of TLKs immediately suggested some plausible mechanisms for their role in DNA repair. We believe that the binding of 9-1-1 and TLK1B to DSBs recruits repair enzymes in conjunction with the chromatin remodeling machinery to create limited repair regions of DNA that is not encumbered by chromatin [9], similar to what has been reported in yeast for the repair of the single DSB at MAT during mating-type switching [81]. We should, however, stress that in such capacity, the role of TLKs as kinases has not been fully elucidated, since for some of these repair functions, expression of the TLK1B-KD was capable of producing effects similar to the catalytically active protein, and in specific reactions of nucleosome assembly, even in the absence of ATP [9, 31, 82]. On the other hand, it seems now clear that the kinase activity of TLKs is very significant in DDR signaling, and most likely during deactivation of the checkpoint. This is the last topic of this paper and is described below.

While studying the gene expression regulatory activities of the translation factor eIF4E, we had originally identified an eIF4E-regulated transcript encoding a protein kinase (TLK1B) that when overexpressed increases radioresistance in mammalian cells [8]. TLK1B is translationally upregulated in response to the presence of DSBs via a mechanism that involves activation of mTOR following that of a PI3K member, likely ATM, which ultimately results in eIF4E stimulation [56]. A rapid response to DNA damage at the translation level is a novel mechanism for cellular survival that has opened new areas of investigation in DSB repair and in damage signaling. Indeed, while transcriptional responses toDNA damage are well known [83], there is no question that the induction of a repair protein (TLK1B) at the translation level would be a far faster response to the injury, and this mechanism of translational activation is now being much more appreciated [84]. While it seemed clear that an increase in TLK1B expression could play some advantageous role during DNA repair, other investigators soon discovered that the kinase activity of TLKs is actually rapidly inhibited during genotoxic stress [85, 86], which initially seemed at odds with our observations for the obvious need for increased levels of TLK1B. Why making more protein kinase if at the same time it is going to be inactive for phosphorylation? Of course this would not include the chaperone function of these proteins [9, 31, 82]. Hence, evidence exists for a strong link between TLKs (both level and activity) and a DNA damage relay [66]. This is inferred by the observation that the kinase activity of TLK1 is inhibited by IR and genotoxins [66]. The inhibition is mediated by ATM via Chk1 by direct phosphorylation of TLK1 at S695 [86]. These findings identified a functional cooperation between ATM and Chk1 in propagation of a checkpoint response mediated by transient inhibition of TLK1, which may regulate processes involved in chromatin remodeling after damage [66]. We believe that the main reason for the cycle of inactivation and then hyperactivation of TLKs, due to the obvious increase in TLK1B expression following genotoxic stress, is to fine-tune chromatin disassembly/reassembly (via Asf1 and/or other histone chaperones) and to mediate the cell cycle checkpoint (particularly its deactivation) via 9-1-1. A model for this was presented in [46] and is repeated here. Asf1 is known to interact with RFC (subunits 2–5) tethered to PCNA, and it is recruited to the replication forks [87]. We propose that after DNA damage, Asf1 is similarly recruited to the lesions to prepare for repair. Here, Asf1 may be instead recruited by the Rad17-RFC clamp-loader, just as Rad9 is, in association with TLK1/1B. After dissociation from RFC [87], the recruited Asf1 is positioned to disrupt the H3/H4 tetramer resulting in nucleosome eviction. As repair progresses, newly synthesized TLK1B induced by DNA damage could lead to dissociation of the Asf1/H3/H4 heterotrimer and promote reformation of the H3-H4 tetramer [82]. Many details remain to be filled in—for instance the role that ATM plays in modulating the two separate activities of TLK1/1B (kinase or chaperone). The association of TLK1B with Asf1 is regulated by its phosphorylation [82]. A possible outcome for the role of ATM-mediated inhibition of TLK1/1B is that the reduction of Asf1 phosphorylation would lead to a more stable association of TLK1/1B-Asf1, instead of a kinetic association involving the ratio of unphosphorylated to phosphorylated Asf1. This could lead to dissociation of the Asf1/H3/H4 trimer. Another question is how the Rad9-mediated checkpoint activation of ATM and ATR may affect the entire pathway and its own association with TLK1/1B and Rad17 [46]. Once TLK1/1B activity is restored after repair, Rad9 may then be rephosphorylated, which could be an important mark for release of the clamp complex and signaling completion of repair and resumption of the cell cycle [46]. Indeed, at least 3 lines of evidence indicate that the TLK kinase activity plays a role in checkpoint establishment and/or its deactivation: (1) the Rad9−/− cells complemented with TLK-KD show a defect in reentering the cell cycle after G2 arrest induced by IR [9]. (2) MM3MG-HO cells expressing the TLK-KD can repair the DSB induced with HO but 10–15% of cells die of apoptosis two days later, which may indicate a defect in deactivating the checkpoint, as supported by the fact that Rad9(S238) phosphorylation is impaired [46]. (3) Mammalian cells treated with a TLK inhibitor + doxorubicin (or IR) arrest preferentially in S phase and die of apoptosis (unpublished results). Hence, we strongly believe that the Rad9-S328 phosphorylation by TLK1 is a key in checkpoint deactivation and suppression of the ATM/ATR-Chk1 signal that is propagated via the clamp/clamp loader and TopBP1—see [53, 88] for two possible models. This would likely involve most types of DNA damage and genotoxic stress and also restart of stalled replication forks—hence the great importance of TLKs for DNA repair and genome integrity.

**Figure 1:**
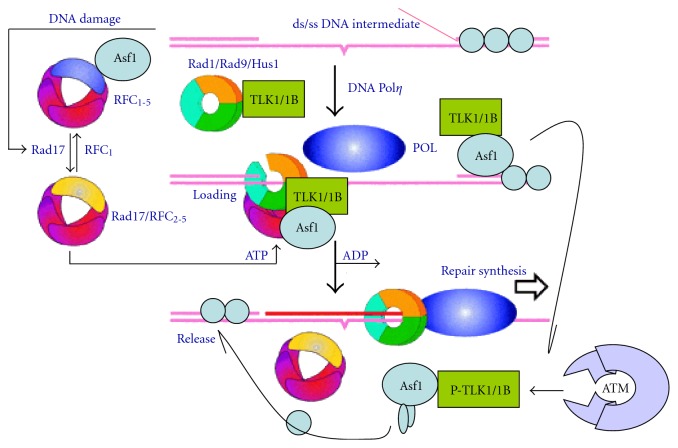
A DNA repair model involving the 9-1-1 complex, Sunavala-Dossabhoy and De Benedetti [9]. Tousled homolog, TLK1, binds and phosphorylates RAD9 and acts as a molecular chaperone in DNA repair. DNA Repair 8(1):87-102.

It would seem obvious that finding inhibitors of TLKs could greatly improve current radio- and chemotherapeutic approaches to cancer treatment. And in fact, silencing TLK1 was highly effective in sensitizing cholangiocarcinoma (a rather incurable disease) cell lines to cisplatin-induced apoptosis [89]. On the other hand, one could envision that exploiting the functions of TLKs in DNA repair could actually produce beneficial effects for normal tissues and organs exposed to the same genotoxic regimens: XRT, radiomimetic chemotherapy, or even daily skin exposure to UV damage. Indeed such cases are being contemplated in our labs, and both gene therapy approaches aimed at sparing salivary glands from the damaging effects of XRT to treat head and neck cancer [90], as well as direct TAT-TLK1 protein delivery to salivary glands [91], have been recently explored with a human clinical trial in sight. Perhaps additional modes of delivery of these proteins, such as a topical skin delivery in a liposomal complex (of either the protein itself or via viral or plasmid gene delivery vehicle), will become feasible in the near future. A model for the participation of TLK in chromatin-remodeling linked to DNA repair is shown in Figure 1.
